# Effect of high-level fine particulate matter and its interaction with meteorological factors on AECOPD in Shijiazhuang, China

**DOI:** 10.1038/s41598-022-12791-4

**Published:** 2022-05-24

**Authors:** Beibei Song, Huiran Zhang, Libin Jiao, Zeng Jing, Honglin Li, Siyu Wu

**Affiliations:** 1 Department of Respiratory and Critical Care Medicine, The Second Hospital of Hebei Medical University/Hebei Key Laboratory of Respiratory Critical Care, No. 215 Heping West Road, Shijiazhuang, 050000 China; 2 Department of Biological Pharmacy, Hebei Medical University, Shijiazhuang, 050000 China; 3 Hebei Far East Communication System Engineering Company, Shijiazhuang, 050000 China; 4 Department of Pharmacy, The Fourth Hospital of Shijiazhuang, Shijiazhuang, 050000 China

**Keywords:** Environmental sciences, Risk factors

## Abstract

Epidemiological evidence of the effect of high-level air pollution and its interaction with meteorological factors on the risk of acute exacerbation of chronic obstructive pulmonary disease (AECOPD) is limited. Daily data on AECOPD cases, air pollutants and meteorological factors were collected from 2015 to 2018 in Shijiazhuang. A distributed lag non-linear model (DLNM) was used to explore the lag and cumulative effect of PM_2.5_ on the risk of AECOPD. The effect of the interaction between PM_2.5_ and meteorological factors on AECOPD was estimated by a generalized additive model (GAM) and a stratification model. A total of 4766 patients with AECOPD were enrolled. After controlling for confounders, each 10 μg/m^3^ increase in PM_2.5_ led to a 5.8% increase in the risk of AECOPD on day lag 0. The cumulative effect of PM_2.5_ on AECOPD risk showed an increasing trend after 3 days. Similar results were observed in both smoking and non-smoking patients. There was an interaction between PM_2.5_ and meteorological factors, and the risk of AECOPD was higher in cold and lower humidity conditions than in other conditions. High-level PM_2.5_ exposure is positively associated with the risk of AECOPD onset, and the effect of PM_2.5_ can be modified by the temperature and relative humidity. Public health guidelines should pay close attention to AECOPD risk under the condition of high-level PM_2.5_ with low temperature or low humidity.

## Introduction

Air pollution is a major contributor to the global burden of disease and was the 4th highest leading risk factor for mortality after high systolic blood pressure, tobacco use, and dietary risks. An estimated 12% of all deaths were attributed to outdoor and indoor air pollution in 2019, and respiratory diseases are linked to an estimated 6.7 million deaths every year around the world^1^.

Chronic obstructive pulmonary disease (COPD) is the leading cause of respiratory disease death in China, resulting in 0.9 million deaths each year and posing huge economic and societal burdens^2^. COPD is a chronic progressive disease, and most COPD hospitalizations are attributed to acute exacerbations^3,4^. Acute exacerbation of chronic obstructive pulmonary disease (AECOPD) is a crucial stage in the natural history of the disease, which leads to the accelerated deterioration of lung function, reduced quality of life, and increased mortality^5–7^. Previous studies have reported that AECOPD patient deaths usually occur in the short term after hospital admission^8,9^. The largest proportion of the total COPD burden has been ascribed to AECOPD in the healthcare system^10^. Ambient air pollutants and meteorological factors are regarded as vital modifiers of the exacerbation process. Mechanistic studies have found that PM_2.5_ can enter distal respiratory tracts due to their small size, resulting in air restriction and severely affecting AECOPD^11^. Recent epidemiological studies have indicated a positive association between low-level PM_2.5_ exposure and the risk of AECOPD^12–15^. Some studies have also suggested that extreme meteorological conditions are associated with the occurrence of AECOPD^16–18^. However, few studies have explored the effect of high-level PM_2.5_ and its interaction with meteorological factors on the development of AECOPD.

Severe air pollution events were reported in Shijiazhuang for a long time during 2015 and 2018. The daily average concentration of PM_2.5_ was occasionally recorded to be over 500 μg/m^3^^19^*.* Our study aimed to evaluate the effect of high concentrations of PM_2.5_ on the risk of AECOPD onset in Shijiazhuang and to investigate the interaction of PM_2.5_ with temperature and relative humidity, using a generalized additive model (GAM). The results are beneficial for developing public health policies and measures to prevent the exacerbation of COPD due to PM_2.5_ and meteorological factors.

## Materials and methods

### Study area

**Figure 1:**
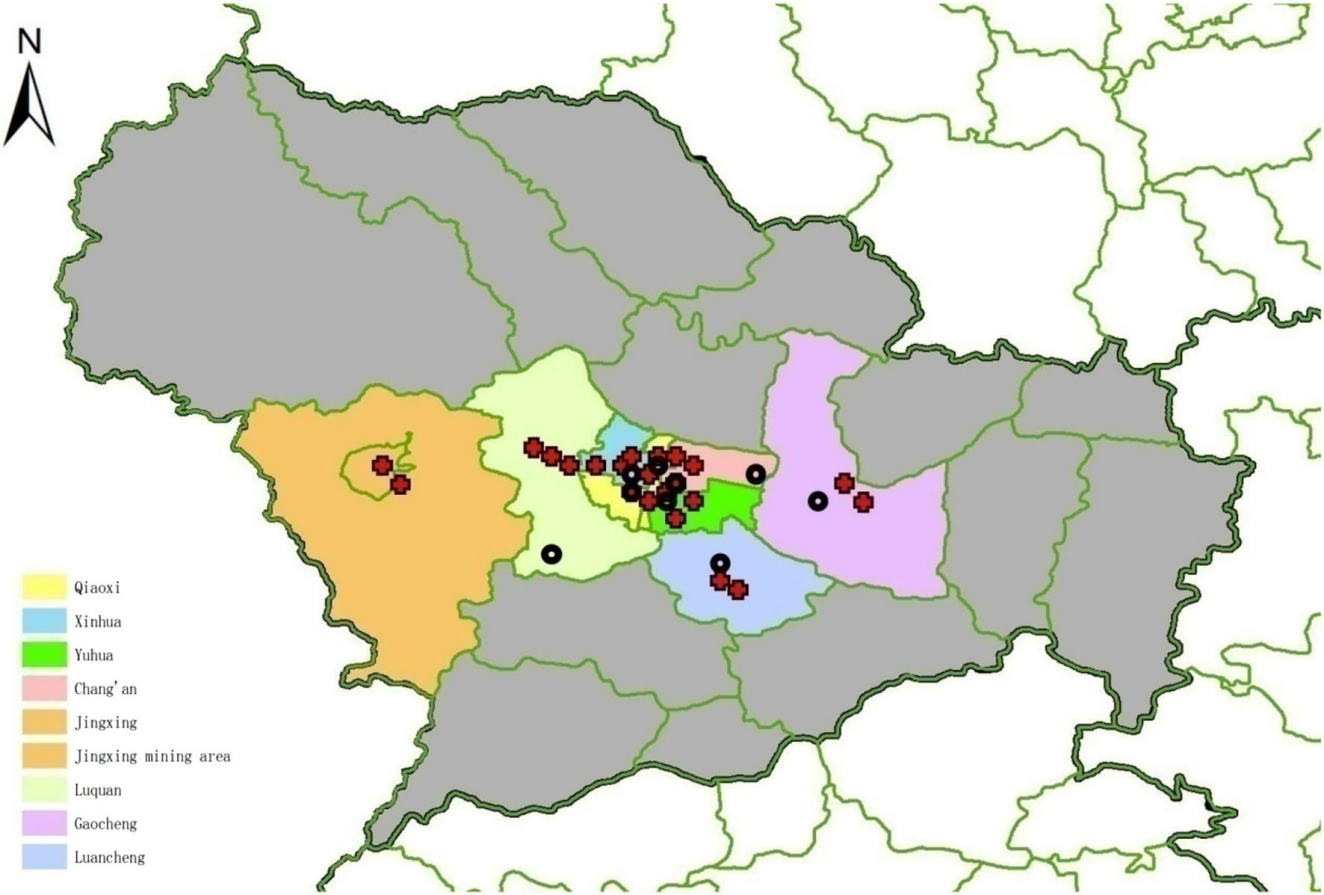
The geographical location of hospitals (red crosses denoting public tertiary hospitals and secondary hospitals) and air quality monitoring stations (black circles) in Shijiazhuang. (ArcGIS 10.2 for Desktop software, Environmental Systems Research Institute Inc., USA, https://www.esri.com).

Shijiazhuang, the capital of Hebei Province, lies in the North China Plain and is one of the largest transport hubs and industrial cities in the North China Plain. The city is located between 113° 30ʹ and 115° 20ʹ N longitude and 37° 27ʹ–38° 47ʹ E latitude and has a sub-humid warm temperate continental monsoon climate and seasonal changes. The annual mean temperature is 12.9 ℃. The 2020 census showed that there were more than 10 million permanent residents in Shijiazhuang. The areas in our study cover more than 60% of the residents of Shijiazhuang. Cars ownership surpassed 2.8 million across the city by the end of 2020 (National Bureau of Statistics, 2021). The geographical locations of hospitals and air quality monitoring stations in Shijiazhuang are shown in Fig. 1.

### Study population

Hospitalization information for AECOPD patients was derived from the electronic medical records of most public tertiary hospitals and secondary hospitals in Shijiazhuang between January 2015 and December 2018. These hospitals accounted for 58% of tertiary hospitals and secondary hospitals and served most of the COPD patients residing in Shijiazhuang. The Electronic Medical System is used to record all patients coming into the hospital who visited a doctor in the emergency department, outpatient department or inpatient department. Our study collected information on patients with AECOPD including dates of hospital admission, principal diagnosis, smoking status, place of residence, and history of diseases. All the patients with primary and secondary diagnoses of AECOPD were included in the analysis. All the AECOPD patients were confirmed according to the standard diagnostic criteria for AECOPD (J44.100 and J44.101) by the 10th Edition International Classification of Diseases. Smoking status was categorized as smokers and non-smokers. The patients were residents of Shijiazhuang city which includes 8 counties, and they lived in Shijiazhuang during 2015–2018. Patients diagnosed, such as cancer, stroke or myocardial infarction, with other severe diseases were not included. This study was conducted according to the guidelines of the Declaration of Helsinki, and all procedures involving human subjects were approved by the Ethics Committee of the Second Hospital of Hebei Medical University. Written informed consent was obtained from all the subjects.

### Data collection

Daily 24-h average concentrations of air pollutants during the same period, including fine particulate matter (PM_2.5_), inhalable particulate matter (PM_10_), sulfur dioxide(SO_2_), nitrogen dioxide(NO_2_), and ozone(O_3_),were obtained from the Hebei Environmental Protection Bureau. We used mass concentration (μg/m^3^) as a unit for the air pollutants, consistent with “national ambient air quality standards” (NAAQS) (GB3095-2012) (http://datacenter.mep.gov.cn/). To adjust for weather conditions, daily average meteorological data including mean temperature (°C) and relative humidity (%) were extracted from the Hebei Meteorological Bureau. All the data on air pollutants and meteorological factors were collected at the national standard weather station in Shijiazhuang.

### Statistical analysis

A descriptive analysis was conducted to describe the temporal distribution of AECOPD cases, air pollutants, and environmental factors.

Previous studies showed that the exposure–response relationship between ambient factors and health outcomes was always non-linear and that the number of daily AECOPD cases approximately followed a quasi-Poisson distribution^20–22^. GAMs have been widely used to explore the association of environmental factors and health outcomes. A GAM with quasi-Poisson link function can control nonlinear confounding factors and over dispersion by nonparametric spline functions^23^. Because of the cumulative and delayed effects of environmental factors on health outcomes, a distributed lag non-linear model (DLNM) was used to estimate the effect of PM_2.5_ and meteorological factors on AECOPD risk^24–26^. PM_2.5_ was applied with a cross-basis function.

Available studies in areas with low-levels of PM_2.5_ showed that the acute influence of ambient air pollutants on the respiratory system always occurred within 6–12 days. In the preliminary analyses, we compared the 7 patterns of the max lag period. We also estimated the degree of model fitting using the determination coefficient (R^2^) and conducted sensitivity analyses. Preliminary analyses showed that a 7-day lag period was the most applicable and robust for our models. Moreover, a recent study in Shanghai reported that the cut-off value of the day prior to acute exacerbation of COPD was 7 days in the warm season^32^. Model diagnostics were further analyzed. Finally, we chose a 7-day lag period for this study. In addition, temperature and relative humidity have previously been shown to be associated with the AECOPD^17,18,27^. Taken together, the formula for the multiple pollutants model was specified as:

$$Log[E(Yt)]=\beta +{\sum }_{p=0}^{7}{\alpha }_{p}{PM2.5}_{t-p}+ s\left(air \,pollutants\right)+s\left(tem\right)+ s\left(hum\right)+ s\left(time\right)+holiday+DOW,$$

where *E(Yt)* is the expected daily count of AECOPD cases on day *t*;* β* is the intercept; and ${\alpha }_{p}$ is the effect estimate of PM_2.5_ *p* days before the onset day of AECOPD. *air pollutants* represents other air pollutants except for PM_2.5_ and was used to adjust for the influence of other pollutants on AECOPD. *tem* and *hum* indicate the daily mean temperature and relative humidity, respectively. *s()* represents the thin plate spline function. *s(time)* was used to control the seasonal and long-term trends. *Holiday* and *DOW* were adjusted for the confounding effect of public holidays and the day of the week because participants had different air pollution exposure levels during the week and during holidays. The optimal degrees of freedom (df) was assessed by generalized cross validation (GCV) criteria. The GCV score can be taken as an estimate of the mean square prediction error based on the leave-one-out cross validation estimation process. The df was automatically selected by R software^28^. Additionally, AECOPD patients were stratified by smoking status to test the modification of tobacco smoking on the risk of AECOPD onset.

The interactions of PM_2.5_ and meteorological factors on AECOPD risk were investigated using a GAM with the thin plate splines function in two steps. First, we used a three-dimensional diagram to explore the potential interactions of PM_2.5_ with temperature and relative humidity. The formula for the model was specified as:

$$ Log(E\left( {Yt} \right) = \beta + s(PM_{2.5} ,\;{\text{meteorological factors}}) + s\left( {air \, pollutants} \right) + s\left( {time} \right) \, + \, holiday \, + \, DOW. $$

Then stratified models were used to examine the effect of PM_2.5_ on AECOPD risk with each level of temperature and relative humidity, namely, low level, medium level and high level, using the cut-point of the 33.3rd and 66.6th percentiles.

R 3.4.3 software (R Foundation for Statistical Computing, Vienna, Austria) was used in all analyses. A two-sided *p* < 0.05 was considered statistically significant.

## Results

### Descriptive analysis

Table 1 shows the main characteristics of AECOPD cases, air pollutants, and meteorological factors in Shijiazhuang between January 2015 and December 2018. A total of 4,766 AECOPD cases were assessed during the study period. The average count of AECOPD cases was 3.26 ± 2.68 per day. The daily average concentrations of PM_2.5_, PM_10_, NO_2_, SO_2_, CO, and O_3_ were 85.4 μg/m^3^, 147 μg/m^3^, 51.6 μg/m^3^, 35.7 μg/m^3^, 1.37 μg/m^3^, and 92.9 μg/m^3^, respectively. The daily mean temperature was 14.5 °C and the relative humidity was 57.4%. The demographic characteristics of the AECOPD patients in Shijiazhuang during 2015–2018 are shown in Supplementary Table 1.

**Table 1:** Descriptive statistics of the AECOPD cases, air pollutants, and meteorological factors in Shijiazhuang during 2015–2018.

	Mean	SD	Min	Percentiles			Max
				25th	50th	75th	
Cases of AECOPD	3.26	2.68	0	1	3	4	23
PM_2.5_ (μg/m^3^)	85.4	73.2	6	39	64	104	621
PM_10_ (μg/m^3^)	147.0	98.0	17	81	126	183	866
NO_2_ (μg/m^3^)	51.6	25.2	9	34	48	64	183
SO_2_ (μg/m^3^)	35.7	30.7	4	15	26	44	259
CO (μg/m^3^)	1.37	1.12	0.1	0.7	1	1.6	10.4
O_3_ (μg/m^3^)	92.9	58.2	2	48	86	128	287
Temperature (℃)	14.5	10.7	− 9.4	4.6	16.3	24.3	35.5
Relative humidity (%)	57.4	20.2	12	42	58	73	99

**Figure 2:**
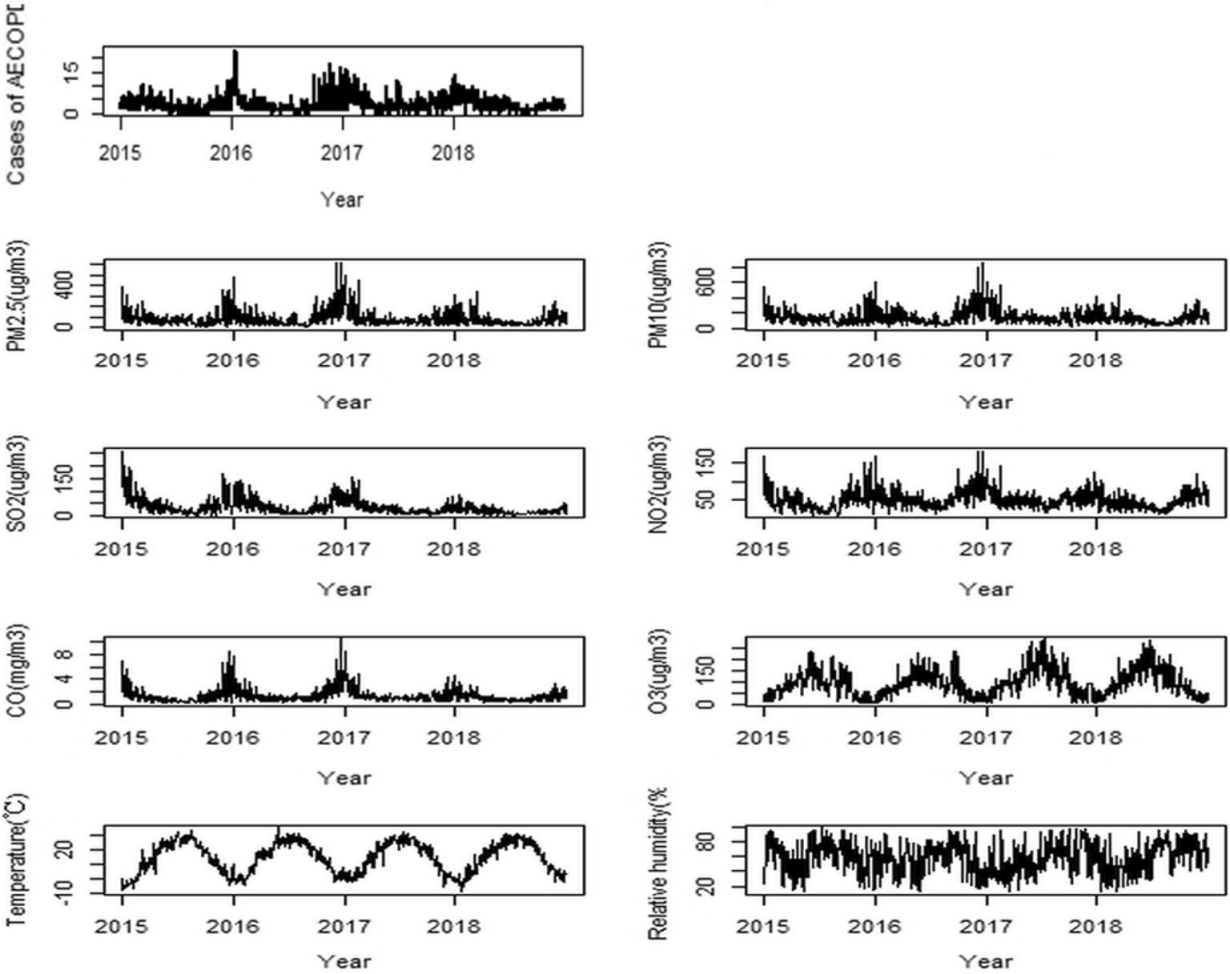
The distribution of the daily AECOPD cases, daily mean air pollutants and meteorological variables in Shijiazhuang, China, 2015–2018.

Figure 2 shows the time-series distributions of the daily AECOPD cases, daily average concentration of air pollutants, daily mean temperature and relative humidity during the study period in Shijiazhuang. The long-term trend of the number of AECOPD cases was mild, and seasonality was found in all series. The number of AECOPD cases increased slightly from 2015 to 2018, with more cases developing in winter and spring.

### Exploratory analysis

**Figure 3:**
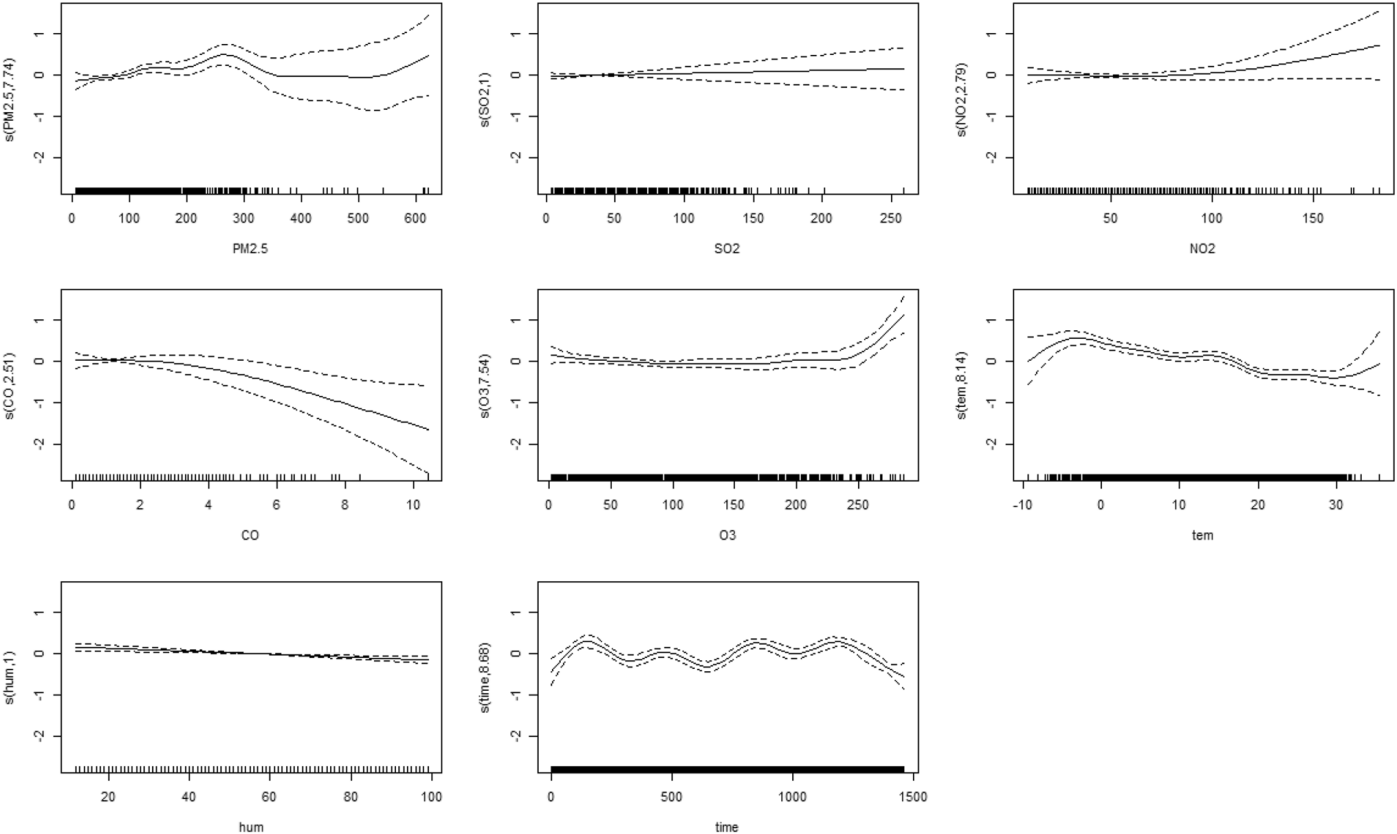
Relationship between air pollutants, meteorological variables and AECOPD cases (*tem*, temperature, *hum* relative humidity). Y-axis: the predicted value of AECOPD cases as the independent variables changed. X-axis: the distribution of variables (including PM_2.5_, other air pollutants, temperature, humidity and time) in the AECOPD patients.

Table 2 presents the Spearman correlation coefficients between AECOPD cases and ambient factors. Daily AECOPD cases were positively and strongly associated with PM_2.5_ (*r*_*s*_ = 0.711) and PM_10_ (*r*_*s*_ = 0.693). Nevertheless, a weak and negative relationship of daily AECOPD cases with temperature and relative humidity was observed. Figure 3 lists the exploratory results from the GAM. A nonlinear relationship of AECOPD cases with PM_2.5_, SO_2_, CO, O_3_ and average temperature was found, while AECOPD cases were linearly associated with NO_2_ and relative humidity. PM_10_was not included in the GAM due to the influence of collinearity between PM_2.5_ and PM_10_ (*r*_*s*_ = 0.981).

**Table 2:** Spearman correlation coefficients of the AECOPD cases with air pollutants and meteorological factors.

	AECOPD	PM_2.5_	PM_10_	SO_2_	CO	NO_2_	O_3_	Temperature	Relative humidity
AECOPD	1.000	0.711*	0.693*	0.433*	0.252*	0.311*	− 0.239*	− 0.450*	− 0.444*
PM_2.5_		1.000	0.981*	0.589*	0.531*	0.642*	− 0.237	− 0.479*	− 0.336*
PM_10_			1.000	0.634*	0.483*	0.443*	− 0.220	− 0.426*	− 0.309*
SO_2_				1.000	0.323	0.423*	− 0.131	− 0.242*	− 0.219*
CO					1.000	0.580*	− 0.220	− 0.212	− 0.166
NO_2_						1.000	− 0.108	− 0.224	− 0.107
O_3_							1.000	0.326*	0.064
Temperature								1.000	0.619*
Relative humidity									1.000

*p < 0.05.

### Regression analysis

The relative risks of AECOPD associated with a 10 μg/m^3^ increase in PM_2.5_ on different lag days are displayed in Table 3. After controlling for other air pollutants, temperature, relative humidity, long-term trends and seasonality, significant associations of PM_2.5_ with AECOPD cases were found in the present analysis. Each 10 μg/m^3^ increase in PM_2.5_ led to a 5.8% increase in the risk of AECOPD on day lag 0. Table 3 also provides the results of the cumulative effect of PM_2.5_ on the development of AECOPD. The results showed that each 10 μg/m^3^ increase in PM_2.5_ led to an 11.4% increase in the cumulative risk of AECOPD on days lag 0–lag 7. The cumulative effect of PM_2.5_ on AECOPD risk showed an increasing trend after 3 days. Similar results were observed in smoking and non-smoking patients.

**Table 3:** Relative risks with 95% CI of AECOPD hospitalizations in Shijiazhuang during 2015–2018 based on 10 μg/m^3^ increases in the fine particulate matter (PM_2.5_) concentration.

	Total patients		Smokers		Non-smokers	
	RR	95% CI	RR	95% CI	RR	95% CI
**Lag effect**						
Lag 0	1.058	1.021–1.095*	1.057	1.014–1.103*	1.065	1.019–1.114*
Lag 1	1.001	0.983–1.019	1.001	0.979–1.022	1.004	0.978–1.030
Lag 2	0.992	0.979–1.005	0.993	0.977–1.009	0.989	0.970–1.009
Lag 3	1.001	0.988–1.014	1.003	0.987–1.018	0.997	0.978–1.017
Lag 4	1.011	0.998–1.024	1.012	0.996–1.027	1.010	0.990–1.030
Lag 5	1.014	1.002–1.027*	1.013	0.997–1.028	1.018	0.999–1.037
Lag 6	1.013	0.996–1.029	1.010	0.989–1.031	1.018	0.993–1.044
Lag 7	1.019	0.997–1.042	1.020	0.993–1.047	1.015	0.982–1.050
**Cumulative effect**						
Lag 0–1	1.059	1.019–1.100*	1.058	1.010–1.108*	1.069	1.019–1.121*
Lag 0–2	1.050	1.009–1.093*	1.051	1.001–1.103*	1.058	1.007–1.113*
Lag 0–3	1.052	1.009–1.096*	1.054	1.002–1.108*	1.056	1.000–1.114*
Lag 0–4	1.063	1.016–1.113*	1.063	1.009–1.126*	1.066	1.004–1.132*
Lag 0–5	1.079	1.028–1.132*	1.079	1.019–1.144*	1.085	1.018–1.158*
Lag 0–6	1.092	1.038–1.149*	1.091	1.026–1.159*	1.105	1.031–1.185*
Lag 0–7	1.114	1.055–1.176*	1.113	1.042–1.187*	1.122	1.040–1.210*

*p < 0.05.

### Interaction analysis

**Figure 4:**
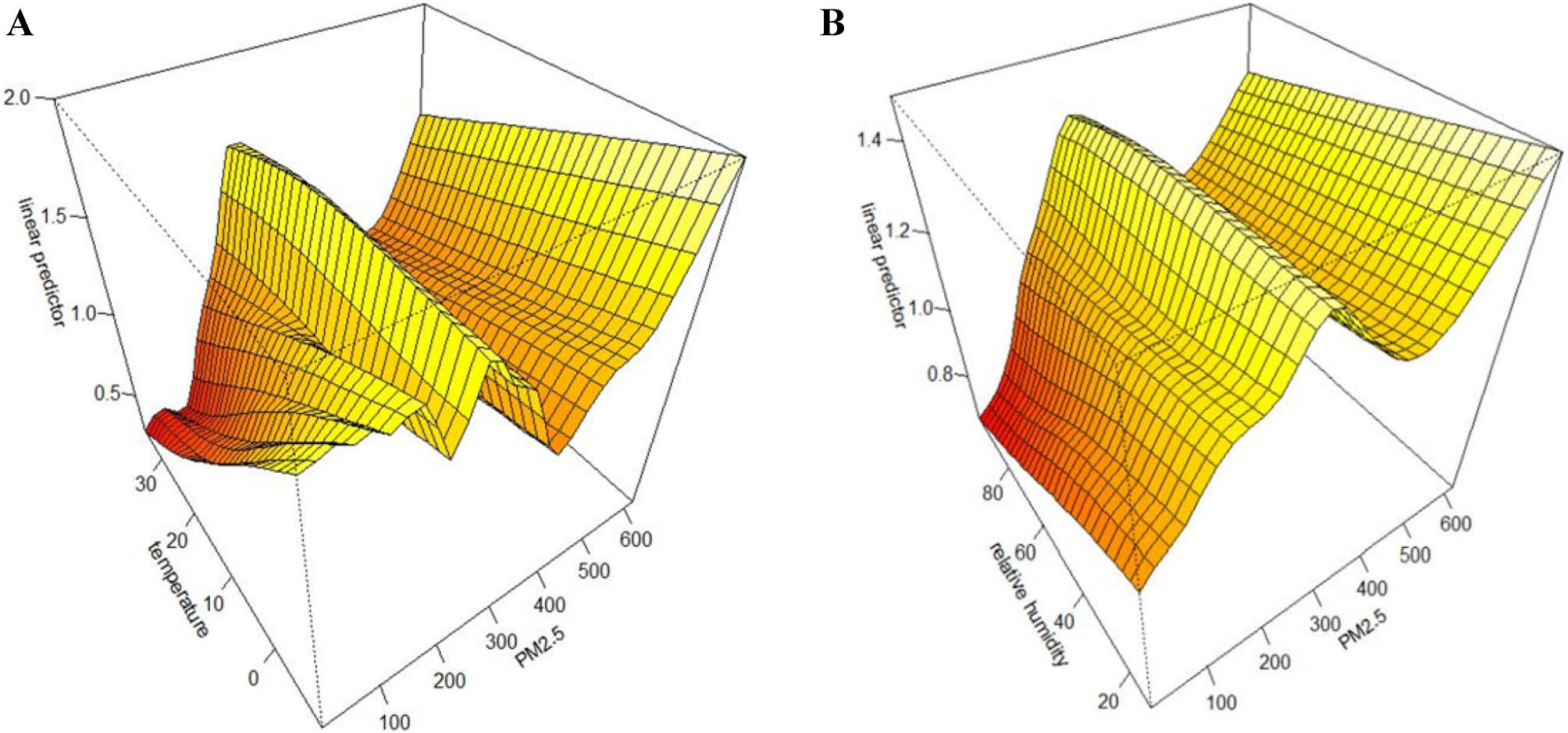
The interaction of PM_2.5_ and meteorological variables on AECOPD cases at present day in Shijiazhuang China, 2015–2018, (**A**) the interaction of PM_2.5_ with temperature; (**B**) the interaction of PM_2.5_ with relative humidity.

**Figure 5:**
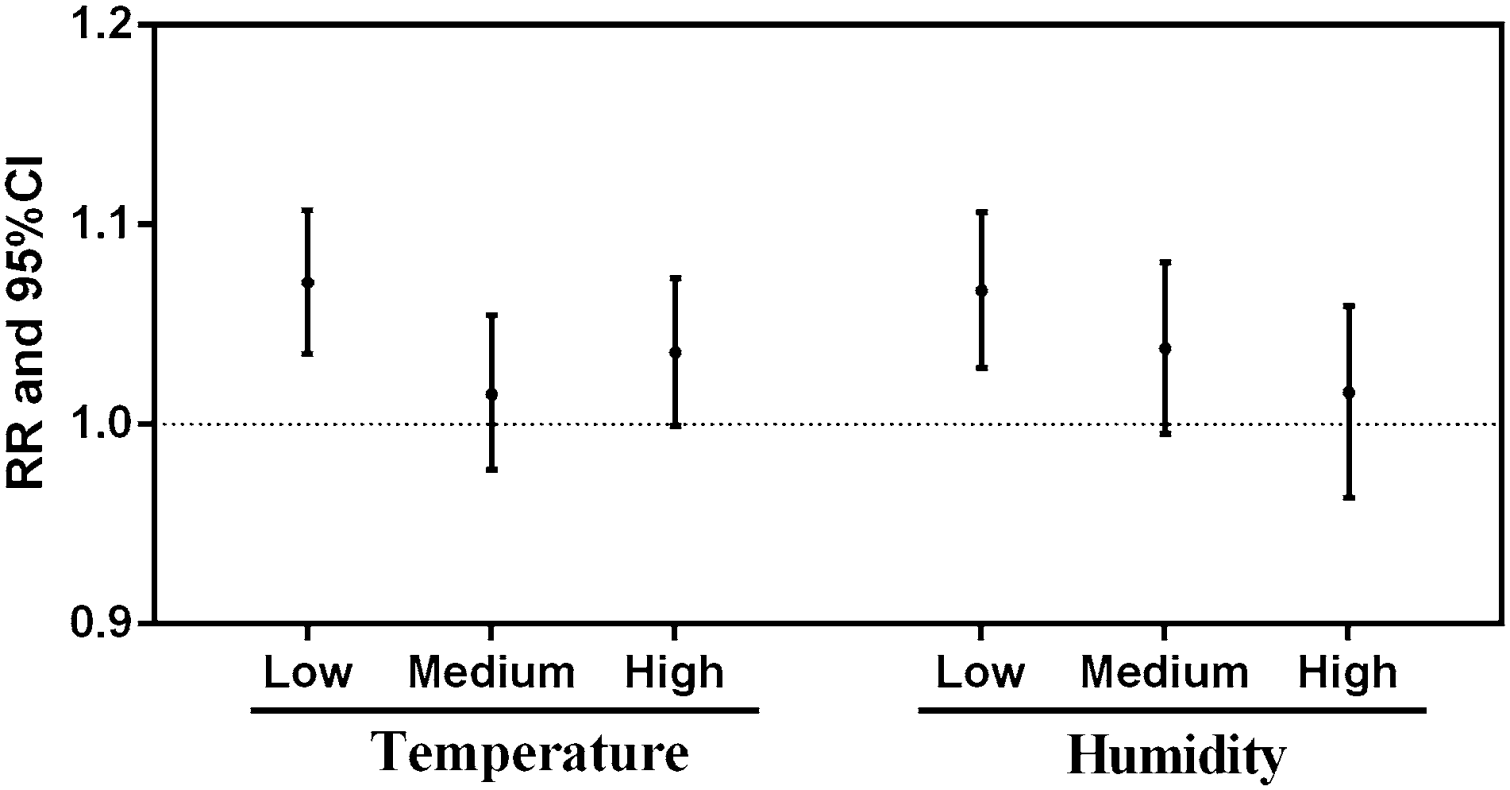
Associations between PM_2.5_ and AECOPD cases based on different levels of temperature and humidity.

Figure 4 displays the interaction of AECOPD cases with different scenarios of temperature and relative humidity using a three-dimensional diagram. After adjusting for the air pollutants, long-term trends and seasonality, the risk of AECOPD on day lag 0 increased when the temperature and relative humidity were low, while the significant effect of PM_2.5_ was not observed for the medium and high levels of temperature and relative humidity. Figure 5 shows that each 10 μg/m^3^ increase in PM_2.5_ led to 7.1% (3.5–10.7%) and 6.7% (2.8–10.6%) increases in the risk of AECOPD on day lag 0 when the temperature and relative humidity, respectively, were low.

### Sensitivity analysis and model diagnostics

Supplementary Table 2 shows the comparisons of 7 patterns max lag period and R^2^. We found that the DLNM was robust for the different max lag times, and the determination coefficient was largest for the 7-day lag pattern (R^2^ = 0.878). Supplementary Fig. 1 shows the result of the model diagnostics. We did not observe obvious autocorrelation in model diagnostics using deviance residual analysis. The model diagnostic results suggested that the DLNMs used in our analyses were robust.

## Discussion

This study explored the effect of high-level PM_2.5_ and its interaction with meteorological factors on AECOPD risk in Shijiazhuang, using time-series analyses including a DLNM and a GAM. Our study indicated that a high level of PM_2.5_ was positively associated with the risk of AECOPD on present day. The results illustrated that PM_2.5_ increased the 7-day cumulative risk of AECOPD. This study also suggested that there was an interaction between high-level PM_2.5_ and meteorological factors on the risk of AECOPD. Low temperature and low humidity strengthened the effect of PM_2.5_ on AECOPD risk.

Convincing time-series studies indicated a positive relationship between PM_2.5_ and the development of AECOPD. Notably, each 10 μg/m^3^ increase in PM_2.5_ concentration on day lag0 of the onset of AECOPD was associated with a 1.05% and 0.8% increase in AECOPD in Yancheng and Shenyang city, respectively^12,13^. Hwang et al. found that a 10 μg/m^3^ increase in PM_2.5_ was associated with a 0.99% increase in the risk of AECOPD on day lag0 in Taiwan^14^. Ko et al. found that a 10 μg/m^3^ increase in PM_2.5_ was associated with a 3.05% increase in AECOPD in Hong Kong^15^. However, former studies were conducted in Chinese cities with a relatively low-level annual mean PM_2.5_ during the study period (45.2 μg/m^3^ in Yancheng, 60 μg/m^3^ in Shenyang, 38.8 μg/m^3^ in Taiwan, and 37.5 μg/m^3^ in Hong Kong). Previous studies with cross-over designs in Europe and the United States were also conducted in areas with a relatively low level of PM_2.5_ in the study period (22.8 μg/m^3^ in Roma, 32.8 μg/m^3^ in Italy and less than 18 μg/m^3^ in America)^29–31^. The differences between the results of our study and those of previous studies may mainly be due to the differences in pollutant concentrations in the different study areas. High-level air pollutants in Shijiazhuang may be attributed to the following reasons. Regarding the terrain, Shijiazhuang is located between the Taihang Mountains and Yanshan Mountains, which form a natural “safe haven” and block the northwestern wind that prevails in the autumn and winter, leading to the accumulation of pollutants that cannot be effectively distributed. However, the cities in other studies were located on a plain (including Yancheng and Shenyang) or close to a sea (including Yancheng, Shenyang, Taiwan and Hong Kong), which helped disperse the pollutants. Moreover, the density of the population in Shijiazhuang is larger than that in cities in other countries. There are more than 10 million permanent residents in 14,464 square kilometer (National Bureau of Statistics, 2021). A larger population density means more potential for pollutant emissions. The use of central heating in winter is another important reason for the high concentrations of PM_2.5_, SO_2_, and NO_2_ in Shijiazhuang. November to March is the annual heating season, and 17.8% of the AECOPD burden has been attributed to PM_2.5_ associated with central heating^19^.

A study in Shanghai suggested that the cut-off value of PM_2.5_ for predicting AECOPD was 83.0 μg/m^3^, and AECOPD may be induced by a high concentration of PM_2.5_^32^. The average annual concentration of PM_2.5_ reached 85.4 μg/m^3^ in our study during 2015–2018. Additionally, this study showed that each 10 μg/m^3^ increase in PM_2.5_ increased the relative risk of AECOPD by 9% with a 3-day cumulative effect in the cold season and 7-day cumulative effect in the warm season. Specifically, each 10 μg/m^3^ increase in PM_2.5_ led to an 11.4% increase in the cumulative number of AECOPD cases 7 days later. The cumulative effect of PM_2.5_ on AECOPD risk represented an increasing trend after three days in our study. Taken together, our findings filled the gap in knowledge of the relationship between a high concentration of PM_2.5_ and the risk of AECOPD.

Cigarette smoking (active and passive) has been widely regarded as an established risk factor for AECOPD^33,34^. The China Pulmonary Health study showed that a two-fold increase in the risk of COPD was associated with smoking exposure of 20 pack-years or more (*OR* 1.95, 95% *CI* 1.53–2.47). However, the results also presented a high prevalence of COPD in never-smokers^35^. Our study investigated the relationship between PM_2.5_ and the number of AECOPD patients stratified by smokers and non-smokers. The relative risk of AECOPD onset was similar among the smoking and non-smoking patients, but the relative risk was slightly higher in the non-smokers (*RR* = 1.065, 95% *CI* 1.019–1.114) than in the smokers (*RR* = 1.057, 95% *CI* 1.014–1.103). This result suggested that the effect of PM_2.5_ on AECOPD among smokers may be stronger than that among non-smokers. Moreover, Xu et al. found that non-smokers may be more sensitive to PM_2.5_ than smokers^36^*.* Therefore, it is necessary to attach equal importance to preventing the onset of AECOPD among smoking and non-smoking COPD patients under conditions of heavy pollution.

In interaction analyses, the results showed that the number of AECOPD cases may increase more under high-level PM_2.5_ and low temperature or low humidity conditions in the study area. Previous studies in Korea and London have suggested that exposure to extremely low temperatures is associated with more severe AECOPD^37,38^. Available studies in Taiwan and Chengdu both show that lower humidity is associated with an increased risk of AECOPD^17,39^. The underlying mechanisms for the increased risk of AECOPD may be explained as follows. On the one hand, a lower temperature could increase the risk of respiratory infections and decrease lung function^40–42^. On the other hand, lower humidity is associated with inducing bronchoconstriction, drying the mucosal membrane along the airway, and therefore increasing the susceptibility to airway bacterial and viral infections^43^. Many studies in Chinese cities, including Shanghai, Yancheng, and Shenyang, have suggested that the association of PM_2.5_ and AECOPD is larger in the cold seasons than in the warm seasons^10,11,30^. As shown in the results of the descriptive analysis, higher PM_2.5_ levels are often accompanied by lower temperature and lower humidity in cold seasons. A study in Chengdu showed that the association of PM_2.5_ and AECOPD risk was significant for temperatures less than 8 °C and humidity less than 60%^17^. Another study in Chengdu also suggested that low temperature and low humidity significantly enhanced the effects of particulate matter on COPD morbidity burden^44^. Hence, COPD patients need to pay more attention to the concentration of PM_2.5_, together with temperature and relative humidity in cold seasons.

Our study is the first to report the risk of AECOPD onset under the condition of a high level of PM_2.5_ and its interaction with meteorological factors using the DLNM. Several limitations of our study should be acknowledged. First, underreporting bias is inevitable for AECOPD cases. Most AECOPD patients are diagnosed with obvious symptoms, whereas patients with mild clinical symptoms may not have been diagnosed and may not have been included in the study. Then, the time-series study missed the personal risk factors that may influence the risk of AECOPD onset, such as social and economic status and living and working environment^45^. Most COPD patients with smoking habits have a long history of smoking and consume a large number of cigarettes per day^46^. Many smokers in the process of quitting return to smoking^35^. Smoking cigarettes permanently and irreversibly damages patients’ lung function even if they quit the smoking habit later in life^47^. Doctors thus regard ever smokers (including ex-smokers) as smokers. Finally, the number of AECOPD cases per day was relatively small, which might have led us to ignore some relationships between PM_2.5_ and AECOPD risk.

## Conclusions

In summary, our study reveals that high-level PM_2.5_ exposure is positively associated with the risk of AECOPD onset in Shijiazhuang, China, and the effect of PM_2.5_ can be modified by the temperature and relative humidity. Non-smoking and smoking patients were both susceptible to the influence of high levels of PM_2.5_. Public health professionals and medical service providers should pay more attention to preventing and controlling a potential increased risk of AECOPD under the condition of high-level PM_2.5_ together with low temperature or low humidity.

## Supplementary Information

Supplementary Information.
