# Supplementary material for: Effect of high-level fine particulate matter and its interaction with meteorological factors on AECOPD in Shijiazhuang, China
Source: Sci Rep. 2022 May 24;12:8711. doi: 10.1038/s41598-022-12791-4 (PMC9130147; doi:10.1038/s41598-022-12791-4)
Supplement: Supplementary file 1 — Supplementary Information. [file 41598_2022_12791_MOESM1_ESM.pdf]

Supplementary table 1. Demographic characteristics of AECOPD patients in Shijiazhuang during 2015-2018.

		Total	Men	Women
**Number**		4766	3843	923
**Age (mean±SD)**		68.8±9.66	68.4±9.46	70.0±9.78
**Smoking habits**				
	Smoker	3085	3042	143
	Non-smokers	1681	801	780

Supplementary table 2. The effect of $PM_{2.5}$ on the risk of AECOPD onset in the different maximum lags patterns.

	**Max lag=6 days**	**Max lag=7 days**	**Max lag=8 days**	**Max lag=9 days**	**Max lag=10 days**	**Max lag=11 days**	**Max lag=12 days**
Lag 0	1.057(1.016-1.099)*	1.058(1.021-1.095)*	1.056(1.019-1.096)*	1.055(1.020-1.096)*	1.056(1.019-1.095)*	1.056(1.018-1.094)*	1.055(1.018-1.097)*
Lag 1	0.999(0.984-1.019)	1.001(0.983-1.019)	1.002(0.991-1.014)	1.002(0.992-1.012)	1.002(0.992-1.011)	1.004(0.995-1.014)	1.005(0.996-1.014)
Lag 2	0.995(0.985-1.006)	0.992(0.979-1.005)	0.994(0.984-1.004)	0.994(0.984-1.003)	0.994(0.985-1.002)	0.997(0.988-1.006)	0.999(0.991-1.007)
Lag 3	1.003(0.991-1.015)	1.001(0.988-1.014)	1.000(0.992-1.007)	0.999(0.993-1.007)	0.999(0.992-1.006)	1.000(0.993-1.007)	1.001(0.994-1.008)
Lag 4	1.006(0.997-1.016)	1.011(0.998-1.024)	1.008(0.999-1.017)	1.008 (0.996-1.016)	1.008(0.996-1.015)	1.006(0.996-1.013)	1.006(0.999-1.012)
Lag 5	1.007(0.993-1.023)	1.014(1.002-1.027)*	1.013(1.005-1.021)*	1.014(1.006-1.021)*	1.010(0.997-1.021)	1.010(0.995-1.020)	1.010(0.998-1.016)
Lag 6	1.022(0.995-1.039)	1.013(0.996-1.029)	1.012(0.993-1.021)	1.013(0.996-1.019)	1.014(0.997-1.021)	1.014(1.007-1.020)*	1.013(1.006-1.019)*
Lag 7		1.019(0.997-1.042)	1.008(0.998-1.019)	1.007(0.998-1.016)	1.009(0.993-1.016)	1.012(0.996-1.017)	1.012(0.996-1.018)
Lag 8			1.009(0.993-1.025)	1.003(0.994-1.012)	1.002(0.994-1.011)	1.006(0.999-1.013)	1.009(1.003-1.014)
Lag 9				1.011(0.996-1.026)	1.002(0.993-1.009)	1.001(0.994-1.009)	1.004(0.998-1.011)
Lag 10					1.016(0.998-1.031)	1.002(0.995-1.009)	1.001(0.994-1.009)
Lag 11						1.016(0.992-1.031)	1.003(0.996-1.009)
Lag 12							1.014(0.999-1.029)
R^2	0.831	0.878	0.792	0.811	0.803	0.810	0.807

*p＜0.05

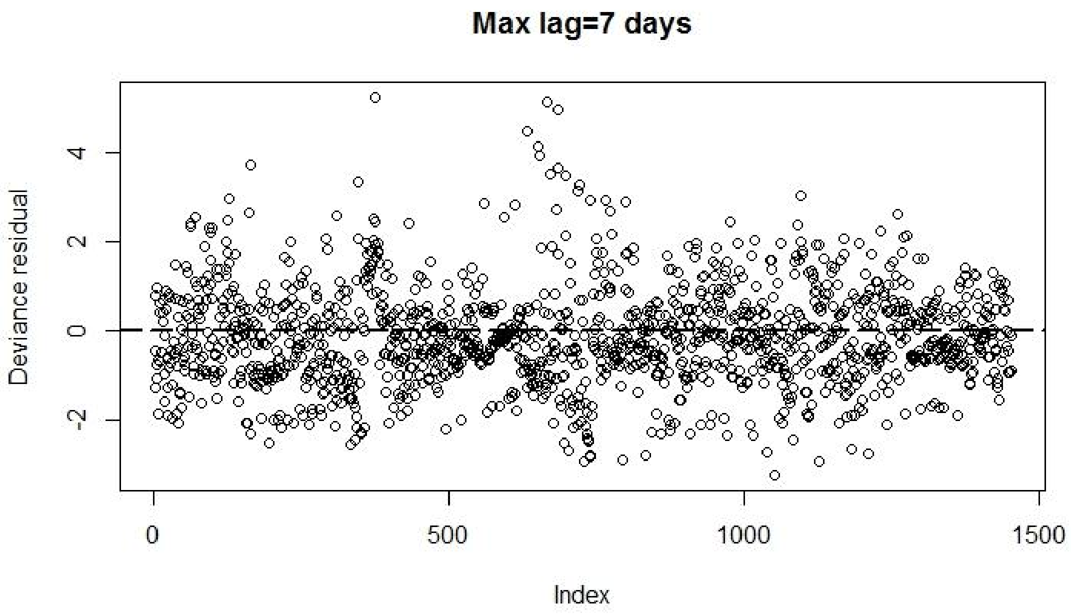

Supplementary figure 1. Model diagnostics of DLNM for 7-day max lag period.
